# Case of Preternatural Labour

**Published:** 1839-01-05

**Authors:** William Denny

**Affiliations:** Ellicott’s Mills


### Case of Preternatural Labour. By William Denny, M.D.

I was requested to visit Mrs. F——, November 11th, 1833, in labour, who was represented to have given birth to one infant of a twin pregnancy. The midwife said that the second fœtus was in malposition, its hand presenting. The uterine efforts were powerful, and occurred with very short intervals of ease. Upon examining with the two first fingers, I found the vagina relaxed, and reached an extremity of the fœtus so high up, and receding so easily on my touch, and yet not advancing by the pains, as to be unable to say whether it was a superior or inferior one. I thought, however, that it was the latter. The liquor amnii of the second ovum had been discharged. With ease, and cautiously, I introduced my hand into the vagina, and made out the presentation as follows, viz.:

Left foot of fœtus, occupying an oblique diameter of the pelvis; heel to left sacro-iliac synchondrosis. Between the fibular edge of that foot, and the pubis of the mother, was the left hand; its dorsum, anterior; thumb, to right extremity of the oblique diameter. Head resting against right acetabulum; os frontis, downward; face directed backward, and to left side of pelvis. When the womb contracted, the presenting parts became wedged in the brim, so as to cause a complete impediment to the progress of the case.

The slightest disturbance by traction at the foot, brought on the action of the uterus; and with it, the wedging of the parts presenting. I adopted the following manœuvre: I passed the fore and middle fingers between the pubis and the foot, and over its dorsum; with the third and fourth above the heel, and against the tendo-achillis, but without disturbing the position even of the *foot* of the fœtus; I lodged the end of my thumb against such point of the head as I found would poise it equally; then pushed slightly but suddenly, so as to cause the head to recede a little, and grasping the foot, so as to make traction on it a continuous motion with the impulse communicated to the head; and changed the position of the fœtus, before the uterine efforts should come on in consequence, and converted that position into a footling case. The manœuvre was successful, and the case terminated well, both for mother and twins.

Maygrier gives some directions for such a case of entanglement, in the "Use of the Repoussoir and Fillet," but the work was not at that time in my possession.

*Ellicott's Mills*, Dec. 30, 1838.

---

*Philadelphia Medical Society.*—At the Annual Election for Officers of the Philadelphia Medical Society, held January 2d, 1839, Dr. Thomas Harris was elected President; Drs. S. Jackson, and R. Coates, Vice Presidents; Dr. Bond, Treasurer and Orator; Drs. B. H. Coates, Warrington, and White, Secretaries; Dr. Johnston, Librarian; Drs. Brewer, Kirkbride, I. Parrish, West, and Patterson, Curators.

